# Tensile Stresses in the Coating with Interlayer under Normal and Tangential Loading

**DOI:** 10.3390/ma15249020

**Published:** 2022-12-16

**Authors:** Adam Stanisław Bajkowski, Rafał Grądzki, Justinas Gargasas, Kristina Bazienė

**Affiliations:** 1 Faculty of Mechanical Engineering, Bialystok University of Technology, 45C Wiejska Street, 15-351 Bialystok, Poland; 2 Faculty of Mechanics, Vilnius Gediminas Technical University, Saulėtekio al. 11, LT-10223 Vilnius, Lithuania

**Keywords:** three-dimensional problem of elasticity, double-layer coating, normal loading, shear loading

## Abstract

In this paper, we obtained the analytic solution of the three-dimensional problem of elasticity concerning non-homogeneous half-space, in which the surface is under normal and tangential loading applied in a circular area. Half-space is composed of the homogeneous body, as well as coatings containing two homogeneous layers: interlayer and top layer. The analysis was carried out in cases where the Young’s modulus of the intermediate layer differed from the Young’s modulus of the substrate and the top layer. We have concentrated on first principal stress analysis. The obtained results can serve as an indication for the design of the composition of coatings in the context of tensile stress.

## 1. Introduction

Coatings improve a surface’s tribological properties, particularly in terms of frictionally cooperative elements. Therefore, the appropriate selection of coatings may cause a decrease in the friction and reduction in the thermal or chemical adverse influence of an environment. Coatings are used when the mechanical contact of elements is unavoidable. Notable examples of coating usage are coatings of switches and plugs (in order to provide good electrical conductivity), tools, bearings, and optical laminas in surfaces subjected to mechanical contact.

In order to evaluate the mechanical properties of a coating, the stress field in the considered medium has to be determined. This stress field arises from contact pressure being applied to its surface. It is crucial to take into account the normal and tangential loading. The formation of cracks should be expected in places of maximum tensile stress. In homogeneous coatings, the maximum value of the *σ*_1_ occurs on the surface in the neighbourhood of the edge of the contact area. This can be observed in articles presenting analytical [1,2] and numerical solutions [3,4] and both ways [5], as well as those describing numerical solutions confirmed by experiments [6] (similar to homogeneous half-space as can be seen in articles from several decades ago [7,8,9]) or the surroundings of the interface between the coating and the base [4,6,10].

Interlayers are used in order to increase the bonding force between the coating and base [3,11,12,13,14]. The occurrence of these layers may also lead to a reduction in tensile stresses near the interface. The two-dimensional problem of the loading of inhomogeneous half-space, composed of two homogeneous layers and a homogeneous body, was considered in [3]. Calculations were carried out using the finite element method. It was emphasised that the location of the maximum value of the characteristics of the stress tensor depends on the relations of Young’s moduli of the components considered half-space, the ratios of the thickness of the components to the characteristic dimension of the contact area, and the coefficient of friction. In article [12], the loading of the coating with an interlayer was considered and the Huber von Mises stresses were calculated using the results of FEM calculations. On this basis, the authors concluded about the appearance of cracks. The axisymmetric problem of loading a body’s surface with double-layer coating was considered in [13,14]. The shape of the indenter (its radius) and the thickness of the coating were analyzed in [15] to obtain the Huber von Mises stress distribution in the medium, and also thus for the moment of initiation of the destruction process.

The scientific research described in the literature was carried out using numerical methods, particularly the finite element method [3,4,6,12].

An analytical solution to the problem of punch movement on a multi-layer substrate was presented in [16]. The results concern the influence of punch speed, coefficient of friction, dimensions and force value on stress distribution. While designing the structure of coatings, a very important task is to ensure appropriate tribological properties. An equally important aspect is to determine the stress value that arises as a result of surface loading [6].

In the present work, we obtain an analytical solution of the three-dimensional problem of elasticity for half-space with a coating composed of two homogeneous layers. The external surface of the non-homogeneous half-space being considered is put under normal and tangential load, applied in a circular area. We focused on the first principal stress analysis.

## 2. Statement of the Problem

**Figure 1:**
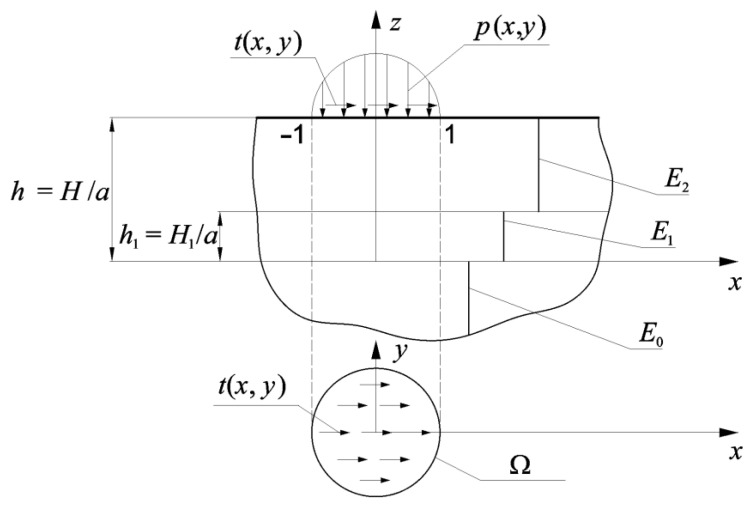
The scheme of the problem.

The subject matter of the considered problem is a homogeneous half-space with the coating of thickness, *H*. The half-space is composed of two homogeneous layers: the interlayer (thickness *H*_1_) and the top layer (thickness *H*—*H*_1_) (Figure 1). The mechanical properties of the body are described by Young’s modulus *E* and by Poisson’s ratio, which is constant in each component of the considered inhomogeneous half-space. Between components of this medium, the ideal mechanical contact condition was satisfied. The surface of the considered half-space is under normal load and related with it by the Amontons–Coulomb’s law tangential load: *t* = *f_p_*, where *f*—coefficient of friction. Loading is applied in a circular area Ω (Figure 1) and has elliptical distribution.

(1) $$p\left(x,y\right)=p_{0}\sqrt{1-x^{2}-y^{2}}$$

where: *x*, *y*, *z*—Cartesian dimensionless coordinates related to radius a of loading circle.

## 3. Mathematical Formulation and Method of Solution

The solution to the problem is based on solving the differential equation:

(2) $$\Delta u_{x}^{\left(i\right)}+d_{i}\frac{\partial \theta_{}^{\left(i\right)}}{\partial x}=0$$

(3) $$\Delta u_{y}^{\left(i\right)}+d_{i}\frac{\partial \theta_{}^{\left(i\right)}}{\partial y}=0$$

(4) $$\Delta u_{z}^{\left(i\right)}+d_{i}\frac{\partial \theta_{}^{\left(i\right)}}{\partial z}=0\;,\;i=0,\;1,\;2$$

with satisfying the boundary conditions:

- loading the surface of inhomogeneous half-space

(5) $$\sigma_{xz}^{\left(2\right)}\left(x,y,z=h\right)=fp\left(x,y\right)H\left(x,y\right)$$

(6) $$\sigma_{yz}^{\left(2\right)}\left(x,y,z=h\right)=0$$

(7) $$\sigma_{zz}^{\left(2\right)}\left(x,y,z=h\right)=-p\left(x,y\right)H\left(x,y\right)$$

- ideal mechanical contact between components of considered half-space

(8) $$u^{\left(i+1\right)}\left(x,y,z=h_{i}\right)=u^{\left(i\right)}\left(x,y,z=h_{i}\right)$$

(9) $$\sigma^{\left(i+1\right)}\left(x,y,z=h_{i}\right)\cdot n=\sigma^{\left(i\right)}\left(x,y,z=h_{i}\right)\cdot n,\;i=0,1$$

- decline the value of components of the stress tensor in infinity:

(10) $$u^{\left(i\right)}\left(x,y,z\right)\to 0\;,\;x^{2}+y^{2}+z^{2}\to \infty ,\;i=0,1,2$$

In Equations (2)–(10), we introduce the following denotations: *θ*^((*i*)^ = div **u**^(*i*)^(*x*, *y*, *z*), *i* = 0, 1, 2; **u**^(*i*)^—dimensionless displacement vector referred to parameter *a*; ***σ*** ^(*i*)^—stress tensor; indexes *i* = 0, *i* = 1, *i* = 2 describe parameters and functions of the state in the base, interlayer and top layer, respectively; *d_i_* = 1/(1–2*ν*_i_); *H*(*x*, *y*)—Heaviside step function (*H*(*x*, *y*) = 1, when (*x*, *y*) ∈ Ω and *H*(*x*, *y*) = 0, when (*x*, *y*) ∉ Ω); *n* = (0, 0, 1); Δ = ∂^2^/∂*x*^2^ + ∂^2^/∂*y*^2^ + ∂^2^/∂*z*^2^—Laplace operator; *h* = *h_1_* + *h_2_*—*z* coordinate of the external surface of inhomogeneous half-space; *h*_0_ = 0—coordinate of the interface between base and interlayer; *h*_1_ = *H*_1_/*a*.

## 4. Method of the Solution

Elasticity Equations (2)–(4) were solved using a two-dimensional Fourier integral transform.

(11) $$\tilde{f}\left(\xi ,\eta ,z\right)=F\left(f\left(x,y,z\right),x\to \xi ,y\to \eta \right)=\frac{1}{2\pi }\int_{-\infty }^{\infty }\int_{-\infty }^{\infty }f\left(x,y,z\right)\exp\left(-ix\xi -iy\eta \right)dxdy$$

The generally solution of Equations (2)–(4), which satisfied boundary conditions (5)–(10), took the form:

(12) $$s^{2}{\tilde{u}}_{x}^{\left(i\right)}=-i\xi {\tilde{\theta }}_{1}^{\left(i\right)}-i\eta {\tilde{\chi }}^{\left(i\right)},\;i=0,1,2$$

(13) $$s^{2}{\tilde{u}}_{y}^{\left(i\right)}=-i\eta {\tilde{\theta }}_{1}^{\left(i\right)}+i\xi {\tilde{\chi }}^{\left(i\right)},\;i=0,1,2$$

(14) $$2{\tilde{u}}_{z}^{\left(0\right)}\left(\xi ,\eta ,z\right)=\left(d_{0}za_{-1}\left(\xi ,\eta \right)+2a_{0}\left(\xi ,\eta \right)\right)\exp\left(sz\right)$$

(15) $${\tilde{u}}_{z}^{\left(i\right)}\left(\xi ,\eta ,z\right)=\sum_{j=1}^{4}a_{4\left(i-1\right)+j}\left(\xi ,\eta \right)\psi_{j}^{\left(i\right)}\left(s,z\right),\;i=1,2$$

where:

(16) $${\tilde{\chi }}^{\left(0\right)}\left(\xi ,\eta ,z\right)=b_{0}\left(\xi ,\eta \right)\exp\left(sz\right)$$

(17) $$2{\tilde{\theta }}_{1}^{\left(0\right)}\left(\xi ,\eta ,z\right)=-\left(\left(2+d_{0}\right)a_{-1}\left(\xi ,\eta \right)+d_{0}sza_{-1}\left(\xi ,\eta \right)+2a_{0}\left(\xi ,\eta \right)s\right)\exp\left(sz\right)$$

(18) $${\tilde{\chi }}_{}^{\left(i\right)}\left(\xi ,\eta ,z\right)=\sum_{j=1}^{2}b_{2\left(i-1\right)+j}\left(\xi ,\eta \right)\chi_{j}^{\left(i\right)}\left(s,z\right),\;i=1,2$$

(19) $${\tilde{\theta }}_{1}^{\left(i\right)}\left(\xi ,\eta ,z\right)=\sum_{j=1}^{4}a_{4\left(i-1\right)+j}\left(\xi ,\eta \right)s\phi_{j}^{\left(i\right)}\left(s,z\right),\;i=1,2$$

Functions from Equations (12)–(19) are defined by the formulas:

(20) $$\chi_{1}^{\left(i\right)}\left(s,z\right)=sinh\left(s\left(h_{i}-z\right)\right),\;\chi_{2}^{\left(i\right)}\left(s,z\right)=cosh\left(s\left(h_{i}-z\right)\right)$$

$$2s\phi_{1}^{\left(i\right)}\left(s,z\right)=\left(2+d_{i}\right)sinh\left(s\left(h_{i}-z\right)\right)+d_{i}s\left(h_{i}-z\right)cosh\left(s\left(h_{i}-z\right)\right)$$

(21) $$2s\phi_{2}^{\left(i\right)}\left(s,z\right)=\left(2+d_{i}\right)cosh\left(s\left(h_{i}-z\right)\right)+d_{i}s\left(h_{i}-z\right)sinh\left(s\left(h_{i}-z\right)\right)$$

$$\phi_{3}^{\left(i\right)}\left(s,z\right)=cosh\left(s\left(h_{i}-z\right)\right),\;\phi_{4}^{\left(i\right)}\left(s,z\right)=sinh\left(s\left(h_{i}-z\right)\right)$$

$$2\psi_{1}^{\left(i\right)}\left(s,z\right)=d_{i}\left(h_{i}-z\right)sinh\left(s\left(h_{i}-z\right)\right)$$

(22) $$2\psi_{2}^{\left(i\right)}\left(s,z\right)=d_{i}\left(h_{i}-z\right)cosh\left(s\left(h_{i}-z\right)\right)$$

$$\psi_{3}^{\left(i\right)}\left(s,z\right)=sinh\left(s\left(h_{i}-z\right)\right),\;\psi_{4}^{\left(i\right)}\left(s,z\right)=cosh\left(s\left(h_{i}-z\right)\right)$$

*i* = 1, 2; *s*^2^ = *ξ*^2^ + *η*^2^; *h*_2_ = *h*; *a*_j_(*ξ*, *η*), *j* = −1, …, 8; *b*_j_(*ξ*, *η*), *j* = 0, …, 4—unknown functions of the parameters of integral transforms, which we determine to satisfy boundary conditions in Equations (5)–(9), written in transform space. Given that a similar conversion was described in [17], we give only several remarks.

Inserting Equations (12)–(19) into Equations (5)–(9), we obtain two independent systems of linear equations to determine functions *a*_j_(*ξ*, *η*) and *b*_j_(*ξ*, *η*). These systems contain 10 and 5 equations, respectively. After solving them and calculating the *a*_j_(*ξ*, *η*) and *b*_j_(*ξ*, *η*) functions, we use the inverse Fourier transform. As a result, we obtained stress tensor components in two-dimensional integral form. Integration was carried out using polar coordinates which were introduced in the integral transform plane. Integrals along the angular coordinate were calculated analytically. Integrals along the radial coordinate in internal points of considered half-space were calculated numerically using the Gaussian quadrature. When calculating the integrals which described stresses at the surface of considered inhomogeneous half-space (z = h), we consider the asymptotic behaviour of the integrand while *s*→∞.

## 5. Results Analysis

Analysis of the obtained relationship indicated that the analytical solution depends on eight dimensionless parameters: *E*_1_/*E*_0_, *E*_2_/*E*_0_, ν_0_, ν_1_, ν_2_, *h*, *h*_1_, *f*. Analysing interlayer influence, we consider cases in which: (1) *E*_1_ < *E*_0_; (2) *E*_0_ < *E*_1_ < *E*_2_; (3) *E*_1_ > *E*_2_. During the calculations we take, respectively: *E*1 = *E*_0_/2; *E*_1_ = (*E*_0_ + *E*_2_)/2; *E*_1_ = 2*E*_2_. Participation of the interlayer’s thickness in the thickness of the whole coating is in the range *h*_1_/*h* = 0.05 ÷ 0.25. The other parameters take the following values: *E*_2_/*E*_0_ = 2 or *E*_2_/*E*_0_ = 4; *h* = 0.4 or *h* = 0.8; *f* = 0; 0.1; 0.25; 0.5; ν_0_ = ν_1_ = ν_2_ = 1/3. In the presented paper, we focus on the tensile stresses applied on the surface of inhomogeneous half-space and the interfaces between its components.

**Figure 2:**
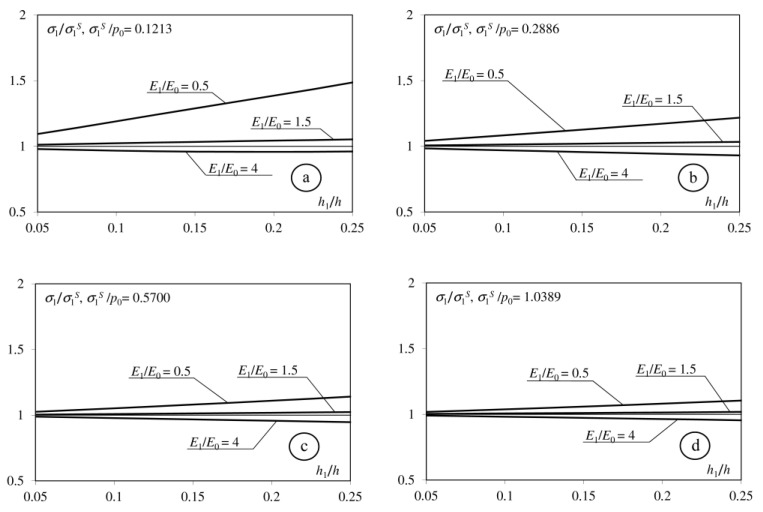
Dependence of the maximum tensile stress on the surface of the considered half-space on the *h*_1_/*h* parameters. (**a**): *f* = 0; (**b**): *f* = 0.1; (**c**): *f* = 0.25; (**d**): *f* = 0.5), *h* = 0.4; *E*_2_/*E*_0_ = 2.

**Figure 3:**
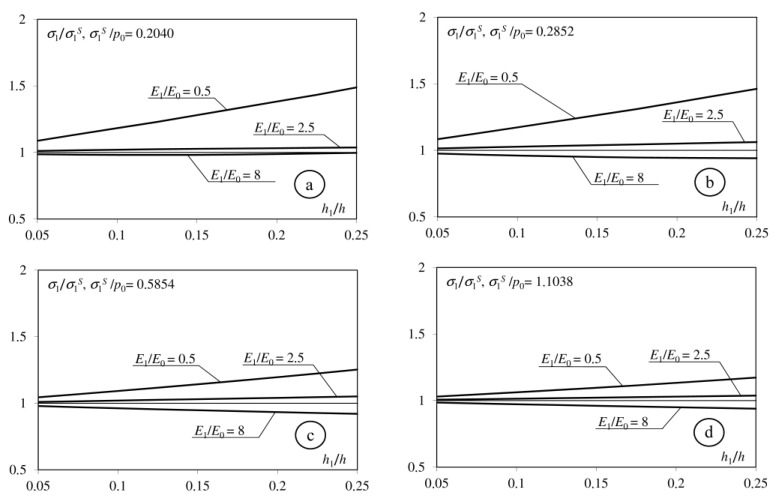
Dependence of the maximum tensile stress on the surface of the considered half-space on the *h*_1_/*h* parameters. (**a**): *f* = 0; (**b**): *f* = 0.1; (**c**): *f* = 0.25; (**d**): *f* = 0.5), *h* = 0.4; *E*_2_/*E*_0_ = 4.

**Figure 4:**
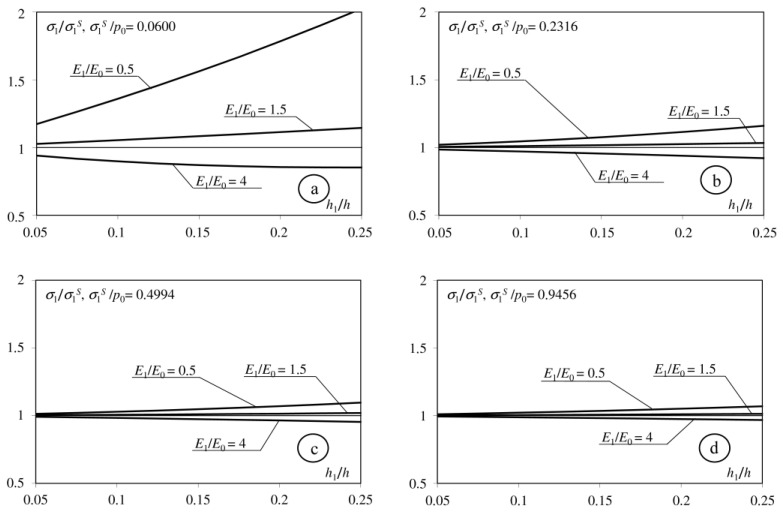
Dependence of the maximum tensile stress on the surface of the considered half-space on the *h*_1_/*h* parameters. (**a**): *f* = 0; (**b**): *f* = 0.1; (**c**): *f* = 0.25; (**d**): *f* = 0.5), *h* = 0.8; *E*_2_/*E*_0_ = 2.

**Figure 5:**
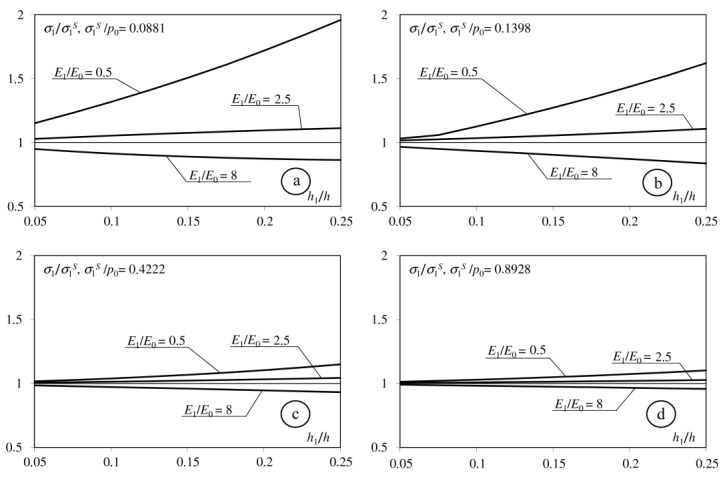
Dependence of the maximum tensile stress on the surface of the considered half-space to the *h*1/*h* parameters. (**a**): *f* = 0; (**b**): *f* = 0.1; (**c**): *f* = 0.25; (**d**): *f* = 0.5), *h* = 0.8; *E*_2_/*E*_0_ = 4.

Figure 2, Figure 3, Figure 4 and Figure 5 shows the dimensionless parameter *σ*_1_/*σ*_1_^S^ in the function of the relative thickness of interlayer *h*_1_/*h*, where: *σ*_1_—maximum tensile stress on the surface of considered half-space, and *σ*_1_^S^—maximum tensile stress in an adequate problem in which the coating does not contain an interlayer. The maximum increase in the tensile stress occurs when the Young modulus of the interlayer is less than the Young modulus of the base (*E*_1_/*E*_0_ = 0.5). This stress increases simultaneously with the parameter *h*_1_/*h*. The interlayer described by the Young modulus *E*_1_ = (*E*_0_ + *E*_2_)/2 causes an insignificant increase in *σ*_1_ stress on the surface. Only introducing the interlayer described by the Young modulus *E*_1_ > *E*_2_ causes a negligible decrease in the tensile stress on the surface. The difference between *σ*_1_ and *σ*_1_^S^ stresses decreases simultaneously when the coefficient of friction increases.

**Figure 6:**
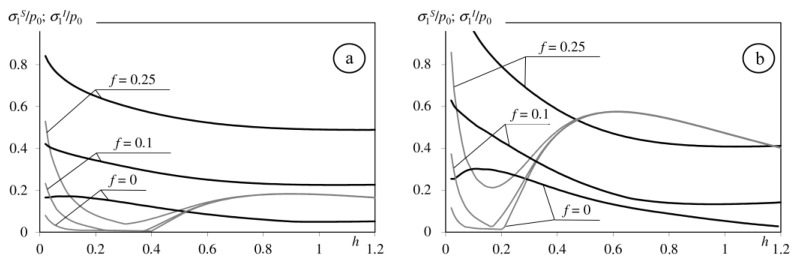
Dependence of the maximum tensile stress on the surface of the considered half-space *σ*_1_^S^ (black lines) and the interface between the homogeneous coating and base *σ*_1_^I^ (grey lines) to coating’s thickness (**a**): *E*_1_/*E*_0_ = 2; (**b**): *E*_1_/*E*_0_ = 4.

**Figure 7:**
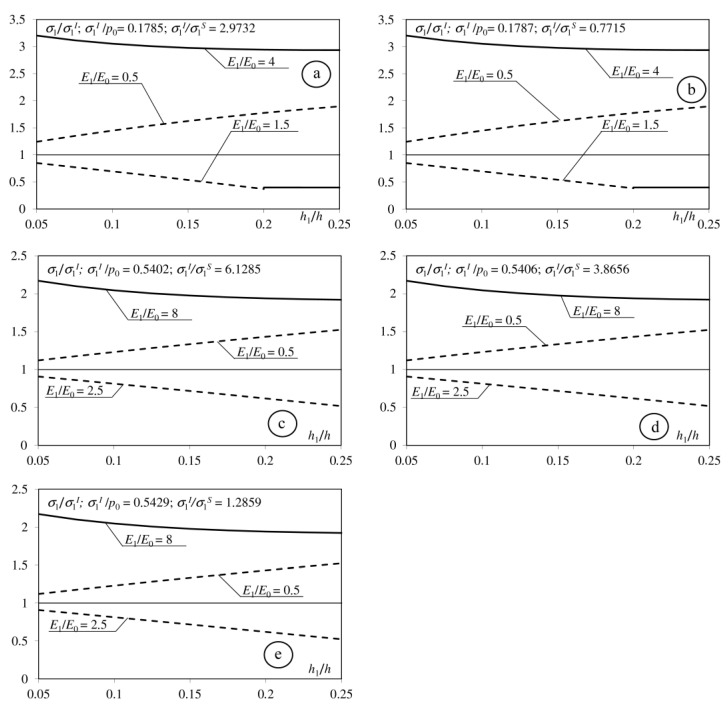
Dependence of the maximum tensile stress on the interface between components of the considered half-space to the *h*_1_/*h* parameters. Dashed line: top layer—interlayer interface; solid line: base—interlayer interface. *h* = 0.8; *E*_2_/*E*_0_ = 2 (**a**): *f* = 0; (**b**): *f* = 0.1); *E*_2_/*E*_0_ = 4 (**c**): *f* = 0; (**d**): *f* = 0.1; (**e**): *f* = 0.25.

Tensile stresses may occur in a medium with a homogeneous coating on the interlayer between the coating and base [4,5,10]. Figure 6 shows the correlation between maximum tensile stress on the surface and analogous stress on the interface between coating and base in a medium with a homogeneous coating in terms of thickness. Tensile stress on the interface takes significant values relative to the corresponding stresses on the surface at a relatively small coefficient of friction and parameter *h* > 0.5 (Figure 6). That is why we analyse the influence of the interlayer to stress distribution near the interface for these parameters (Figure 7). This figure shows the ratio of the stresses *σ*_1_/*σ*_1_^I^ in the function of the thickness of interlayer *h*_1_/*h* (*σ*_1_—maximum tensile stress on the interfaces between components of the considered medium with double-layered coatings, *σ*_1_^I^—maximum tensile stress on the interface between coating and base in the medium with the homogeneous coating (Figure 6)). If the *σ*_1_ stress take larger values on the interface between base and interlayer, then the graph is a continuous line; when larger values occur on the top layer-interlayer interface, the line is dashed. Introducing the interlayer, which is described by a Young modulus lower than base *E*_1_ < *E*_0_ (*E*_1_ = *E*_0_/2) and larger than top layers modulus *E*_1_ > *E*_2_ (*E*_1_ = 2*E*_2_), results in the increase in stresses on the interface relative to corresponding stresses in the medium with homogeneous coating. A decrease in the stresses occurs when the Young modulus of the interlayer has a value between the top coat and base moduli: *E*_0_ < *E*_1_ < *E*_2_ (*E*_1_ = (*E*_0_ + *E*_2_)/2).

## 6. Conclusions

In the presented work, the analytical solution to the three-dimensional problem of elasticity was considered. The research object is the half-space whose surface is under normal and tangential load. The half-space comprises a homogeneous body and coating, including the top layer and interlayer.

Based on the stress analysis, introducing an interlayer with a Young modulus of *E*_1_ < *E*_0_ causes a significant increase in the *σ*_1_ stress on the surface of the inhomogeneous half-space. When *E*_1_ > *E*_2_, the tension stresses on the interface increase. Therefore, the choice of interlayer in which Young modulus is in range (*E*_0_, *E*_2_) seems to be optimal. Introducing this layer may considerably reduce the tensile stresses near the interface between components of the considered medium. The increase in the stresses on the surface is insignificant. The test results can help in the selection of the composition of the cover in terms of minimizing the occurrence of tensile stress in it.
